# Structural characteristics and diversity of the rhizosphere bacterial communities of wild *Fritillaria przewalskii* Maxim. in the northeastern Tibetan Plateau

**DOI:** 10.3389/fmicb.2023.1070815

**Published:** 2023-02-17

**Authors:** Zhijia Cui, Ran Li, Fan Li, Ling Jin, Haixu Wu, Chunya Cheng, Yi Ma, Zhenheng Wang, Yuanyuan Wang

**Affiliations:** ^1^College of Pharmacy, Gansu University of Chinese Medicine, Lanzhou, Gansu, China; ^2^Northwest Collaborative Innovation Center for Traditional Chinese Medicine Co-Constructed by Gansu Province & MOE of PRC, Lanzhou, Gansu, China

**Keywords:** *Fritillaria przewalskii* Maxim., natural population, rhizosphere soil, bacterial community, characteristics and diversity, northeastern Tibetan Plateau

## Abstract

**Introduction:**

*Fritillaria przewalskii* Maxim. is a Chinese endemic species with high medicinal value distributed in the northeastern part of the Tibetan Plateau. *F. przewalskii* root-associated rhizosphere bacterial communities shaped by soil properties may maintain the stability of soil structure and regulate *F. przewalskii* growth, but the rhizosphere bacterial community structure of wild *F. przewalskii* from natural populations is not clear.

**Methods:**

In the current study, soil samples from 12 sites within the natural range of wild *F. przewalskii* were collected to investigate the compositions of bacterial communities *via* high-throughput sequencing of 16S rRNA genes and multivariate statistical analysis combined with soil properties and plant phenotypic characteristics.

**Results:**

Bacterial communities varied between rhizosphere and bulk soil, and also between sites. Co-occurrence networks were more complex in rhizosphere soil (1,169 edges) than in bulk soil (676 edges). There were differences in bacterial communities between regions, including diversity and composition. Proteobacteria (26.47–37.61%), Bacteroidetes (10.53–25.22%), and Acidobacteria (10.45–23.54%) were the dominant bacteria, and all are associated with nutrient cycling. In multivariate statistical analysis, both soil properties and plant phenotypic characteristics were significantly associated with the bacterial community (*p* < 0.05). Soil physicochemical properties accounted for most community differences, and pH was a key factor (*p* < 0.01). Interestingly, when the rhizosphere soil environment remained alkaline, the C and N contents were lowest, as was the biomass of the medicinal part bulb. This might relate to the specific distribution of genera, such as *Pseudonocardia*, *Ohtaekwangia*, *Flavobacterium* (relative abundance >0.01), which all have significantly correlated with the biomass of *F. przewalskii* (*p* < 0.05).

**Discussion:**

*F. przewalskii* is evidently averse to alkaline soil with high potassium contents, but this requires future verification. The results of the present study may provide theoretical guidance and new insights for the cultivation and domestication of *F. przewalskii*.

## 1. Introduction

*Fritillaria* is a genus of herbaceous perennial plants that contains 130–165 species (Hao et al., 2013). *Fritillaria przewalskii* Maxim. is the common species of the genus, and its bulbs possess moistening dryness and clearing lung heat effects as a source of the Chinese traditional cough medicine Fritillariae Cirrhosae Bulbus (Ma et al., 2020). Alkaloids are considered to possess pharmacological activity that reduces lung injury caused by various factors, and are the largest class of photochemical components in *F. przewalskii* (Majnooni et al., 2020). Numerous recent studies indicate that *F. przewalskii* has potential antineoplastic, anti-inflammatory, and anti-tumor effects (Wang Y. et al., 2021). Consequently, the medicinal value of *F. przewalskii* is extremely high. Wild *F. przewalskii* is distributed in high altitude areas in the northwestern part of the Tibetan Plateau, but it has been overharvested and is now classified as an endangered species (Chen T. et al., 2020). To meet market demand, wild *F. przewalskii* are gradually being replaced by cultivated products (Li et al., 2009). Due to the specialized wild habitat of *F. przewalskii*, and its unique soil requirement, cultivated products will likely be of poorer quality than wild *F. przewalskii* (Ma et al., 2020). Several common soil-borne diseases, such as wilt, sclerotinia rot, and root rot, are also causes of production failure (Baayen et al., 2001; Song et al., 2016; Qiao et al., 2022). There is an urgent need to ensure the healthy growth of *F. przewalskii*, and improve the quality of *F. przewalskii* circulating in market.

The rhizosphere is the main area of material exchange between the plant and the soil ecosystem, and it contains a diversity of microorganisms (Raaijmakers et al., 2009). Previous studies suggest that plant-associated microbial function confers environmental fitness advantages to host plants that are crucial to supporting plant growth and health in the natural environment (Mendes et al., 2011; Richardson and Simpson, 2011; Fitzpatrick et al., 2019). For example, enrichment of specific functional microbial taxa can improve plant drought resistance (Fitzpatrick et al., 2019), increase the availability of nutrients in soil (Koranda et al., 2011), and regulate the plant’s immune system and enhance resistance to pathogens (Lebeis et al., 2015). Hence, the growth of plants highly depend on their interactions plant-associated microbes (Chaparro et al., 2014; Kong et al., 2019). The abilities of wild progenitors to resist biotic and abiotic stresses are better than cultivated plants (Qu et al., 2020; Gutierrez and Grillo, 2022). These properties, formed under interaction with environment, are regulated by the associated rhizosphere microbiome (Mantelin and Touraine, 2004). Accordingly, understanding the structural characteristics and functional compositions of the rhizosphere microbial community in a wild habitat provides a scientific reference for its artificial cultivation. To date however, the rhizosphere microbial communities of wild *F. przewalskii* have remained elusive.

Rhizosphere microbial communities are dynamically changing due to various influences, which in turn affect material circulation and energy flow in the rhizosphere environment, and ultimately the growth and development of plants (Eisenhauer et al., 2012). Advanced technologies such as culture-independent high-throughput sequencing techniques can reveal the structure of rhizosphere microbial communities, and facilitate investigation of plant–soil-microbiome interactions (Choi et al., 2021). Changes in the diversity and composition of rhizosphere microbial communities are often associated with the host plant and its environment, including root secretions, plant growth stages, soil type, and physicochemical properties (Chaparro et al., 2014; Xue et al., 2018; Broadbent et al., 2021). Host plant associated soil microhabitats influence rhizosphere microorganism enrichment (Xiong and Lu, 2022). For example, in recent studies in lilies the rhizosphere microbial community composition differed significantly in different habitats (Xie et al., 2021). This can be explained by the fact that root secretions and soil physicochemical properties vary in different microhabitats, thus selectively assembling specific microbiomes. Due to the specialized native habitat of *F. przewalskii*, there is currently no comprehensive understanding of the rhizosphere microbial communities involved in its growth.

*Fritillaria przewalskii* is distributed at 2,400–4,300 m altitude in the alpine grasses of the northeastern part of the Tibetan Plateau. As the highest and largest plateau on Earth, the Tibetan Plateau has a unique alpine ecosystem with complex and diverse microbiota (Chen et al., 2021; Xu et al., 2021). The habitat is extremely vulnerable to natural influences, and human activities including over-harvesting, which have now resulted in *F. przewalskii* being declared endangered (Chen et al., 2013; Jin et al., 2019). Numerous studies indicated that knowing more about rhizosphere microbiota patterns may enhance the effectiveness of measure designed to assist the adaptation of *F. przewalskii* to the environment, and resist stress (Mahoney et al., 2017; Jiao et al., 2022). In particular, rhizosphere microbes can improve tolerance of *Fritillaria* to common diseases, such as wilt and root rot diseases, which can reduce crop yields (Baayen et al., 2001; Song et al., 2016). Nevertheless, to our knowledge, there is no information available on wild *F. przewalskii* root-associated microbial communities, specifically comparative studies of different natural growth areas.

In this current study, 16S rRNA gene amplicon sequencing of soil samples collected from 12 different natural growth areas was conducted to investigate the compositions and functional phenotypes of the soil bacterial communities of wild *F. przewalskii*. The aims were to (1) investigate differences in bacterial community composition and diversity between rhizosphere and bulk soil samples, as well as among sites; (2) elucidate the functional phenotypes of common microorganisms at different sites, and specific microorganisms at each site; (3) indentify suitable soil environments for *F. przewalskii* combined with phenotypic characteristics of *F. przewalskii* at different sites; and (4) provide a comprehensive understanding of the key factors affecting rhizosphere bacterial communities. The results of the study shed new light on the microbial composition of *F. przewalskii*, and may provide theoretical guidance and new insights for resource conservation, artificial cultivation and domestication of *F. przewalskii*.

## 2. Materials and methods

### 2.1. Soil collection

**Figure 1:**
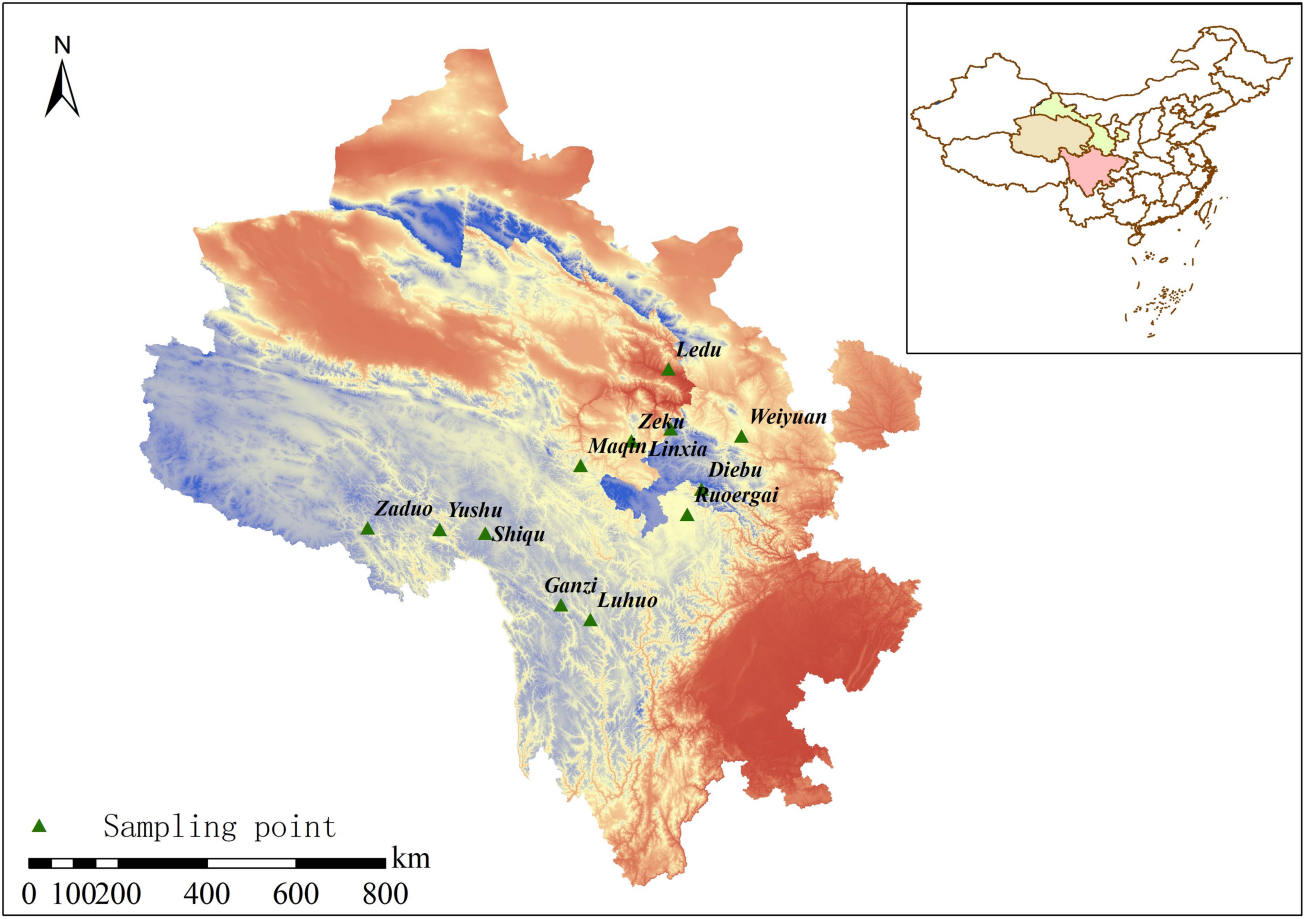
Distribution of sampling points.

Soil samples were collected from 12 areas within the range of wild *F. przewalskii* between late July and early August, including Weiyuan County (WY), Linxia County (LX), Diebu County (DB), Ruoergai County (RG), Ganzi Tibetan Autonomous Prefecture (GZ), Shiqu County (SQ), Luhuo County (LH), Zeku County (ZK), Yushu City (YS), Zaduo County (ZD), Maqin County (MQ), and Ledu District (LD) (Figure 1). At each site, five *F. przewalskii* plants were randomly selected by five-point sampling method (Xie et al., 2021). We only chose plants that had grown for more than 3 years. Rhizosphere soil was carefully collected gently by removing the soil stuck to roots and bulbs using a brush, while bulk soil was collected around the root system with a sterile shovel approximately 5 cm away from each removed plant to a depth of 15 cm (Zhang Z .C. et al., 2021). Each of the five soil samples from the same site was homogenized then sub-sampled three times, resulting in a total of 72 samples (36 rhizosphere and 36 bulk soils). After collecting soil samples, all selected plants were divided into leaves, stems, bulbs, and roots. Leaf length and width, stem length and diameter, bulb horizontal and vertical dimensions, and root length were measured using vernier calipers (Ma et al., 2020). Single bulb fresh weight was measured with an electronic scale. Soil samples were divided into two parts. One was immediately frozen *via* liquid nitrogen submersion, transported to the laboratory, and stored at −80°C for microbial analysis, and the other was air-dried for soil characterization.

### 2.2. Soil physiochemical analysis

The samples used for soil physiochemical analysis were all rhizosphere soils. Soil pH was measured in a soil solution at a soil: water ratio of 1:5 using a pH meter. Soil was pretreated with K_2_Cr_2_O_3_ and mixed with H_2_SO_4_ to oxidize organic carbon. The combustion method was then used to determine the soil organic carbon (SOC) level (Nishanth and Biswas, 2008; Kim et al., 2021). Soil total nitrogen (TN) was determined *via* the micro-Kjeldahl method using an automatic Kjeldahl instrument (K8400, FOSS Corporation, Denmark) (Bremner, 1996). Nessler reagent spectrophotometry was used to measure soil ammonium-nitrogen (NH^4+^-N). HCl was used to extract soil total phosphorus (TP) and 0.5 mol/L NaHCO_3_ was used to extract available phosphorus (AP). Both were quantified by the molybdenum blue method (Fan et al., 2018; Liu et al., 2020). Total soil potassium (TK) was measured using an atomic absorption spectrophotometer (PinAAcle 900 T, United States) (Wang and Huang, 2021).

### 2.3. DNA extraction and sequencing

Total soil DNA was extracted from rhizosphere and bulk soil *via* a DNeasy PowerSoil Pro kit (Qiang, Germany). For bacterial community diversity and composition, the V3-V4 hypervariable region of the 16S rRNA gene was amplified with primers 338F (5′-ACTCCTACGGGAGGCAGCAG-3′) and 806R (5′-GGACTACHVGGGTWTCTAAT-3′) (Caporaso et al., 2011). The PCR reaction (50 μL) reagents used were 2 μL template DNA, 2 × 25 μL Phanta Max Master Mix, and 10 μmol/L of each primer. The PCR conditions were predenaturation temperature 94°C (30 s), denaturation temperature 94°C (30 s), annealing temperature 55°C (45 s), and extension temperature 72°C (45 s), then after 30 cycles extension was terminated at 72°C for 10 min. Sequencing was performed offsite by BGI Genomics Co. Ltd. (Shenzhen, China) using an Illumina MiSeq platform.

### 2.4. Sequence processing

After quality-filtering using QIIME (v.1.7.0) (Caporaso et al., 2010), chimeric sequences were removed using the UCHIME procedure (Edgar et al., 2011). Raw sequence reads were spliced using FLASH software (Magoč and Salzberg, 2011), then clustered into operational taxonomic units (OTUs) at a 97% similarity level using USEARCH software (v7.0.1090_i86linux32) (Edgar, 2013). Taxonomy assignments were done using RDP classifier software (v1.9.1) at the 80% confidence level against the Greengenes database (DeSantis et al., 2006). Upset diagram was drawn using the R “UpSetR” package (v4.0.4) (Andrysik et al., 2017). Alpha diversity indices such as the Chao index, Ace index, Shannon index, and Simpson index were calculated using Mothur (Schloss et al., 2009). Significant differences in bacterial communities were visualized *via* partial least squares discriminant analysis (PLS-DA) using SIMCA software (v14.1) (Yang et al., 2021) and the unweighted pair group method with arithmetic averages (UPGMA) using the R “vegan” package (Nazish et al., 2017). Species relative abundance was tabulated at each taxonomic level using Qiime software (Caporaso et al., 2010), then plotted with OriginPro 2022. Linear discriminate analysis (LDA) was performed using the R “topicmodels” package (Zhang Y. et al., 2021) to determine the different distributions of bacterial genera at the phylum level in samples. A heatmap of relative abundance at the genus level was generated using the R “pheatmap” package (Mortensen et al., 2016). Different genera and dominant genera in samples were identified and visualized the results by circus plot using the R “circlize” package (Yang et al., 2016).

Co-occurrence networks were inferred for rhizosphere and bulk soil to assess the complexity of interactions among bacterial taxa based on the Spearman’s correlation coefficient, calculated using the “Hmisc” package in R (Huang et al., 2018). OTUs found in at least half of the samples were selected to establish co-occurrence networks. The nodes in the network represented OTUs, while the edges represented significant positive or negative correlations between two nodes. The network was visualized using Gephi 0.9.6 (*r* [absolute value] > 0.6, *p* < 0.01). Measurement properties such as numbers of nodes and edges, average degree, and average clustering coefficient were calculated using Gephi (Tu et al., 2019).

### 2.5. Statistical analysis

Significant differences were analyzed *via* one-way ANOVA in SPSS (v26.0) (Anderson, 2001) with variables including plant phenotypic characteristics, soil characteristics, and alpha diversity indices. Principal component analysis (PCA) was performed using “ggplot2” and “FactoMineR” packages in R to investigate the distribution of soil characteristics and separate rhizosphere bacterial communities in the samples (Brückner and Heethoff, 2017). Bacterial community composition of rhizosphere and bulk soil samples was analyzed based on the nonmetric multidimensional scaling (NMDS) of the Bray–Curtis dissimilarity using the R “vegan” package (Goslee and Urban, 2007). To identify significant effects of plant phenotypic characteristics and soil characteristics on bacterial communities, a bubble plot was drawn using the R “ggplot2” package (Dussud et al., 2018), and redundancy analysis (RDA) performed using CANOCO (v5.0) (Wu et al., 2021). Correlation analysis was performed using the Mantel test in the R “vegan” package and plotted with the “ggcor” package in R (Xue et al., 2021).

## 3. Results

### 3.1. Plant phenotypic characteristics

*Fritillaria przewalskii* had different phenotypic characteristics in different habitat (Supplementary Table S1). All characteristics except for stem diameter and root length, differed significantly across some sites (*p* < 0.05). In general, aboveground traits were best at the ZK site, whereas at the DB and LD sites they were inferior. *F. przewalskii* at the ZK site was above 45 cm in height, which was significantly higher than that at the DB, SQ, YS, ZD, and MQ sites (all below 30 cm), whereas there were no significant differences detected between other sites (*p* < 0.05). Leaf traits varied little. Leaf length and width data indicated that plants at the ZK site had significantly larger leaf area than those at the LX, DB, and LD sites (*p* < 0.05). Bulb traits differed greatly, and were best at the MQ site. The bulb weight of *F. przewalskii* at the MQ site was 10.87 times greater than that at the lightest site (DB), and it differed significantly from all regions except the ZK site (*p* < 0.05). The bulb weight of *F. przewalskii* at ZK also differed significantly from those at DB, RG, YS, and LD sites (*p* < 0.05).

### 3.2. Soil characteristics

**Figure 2:**
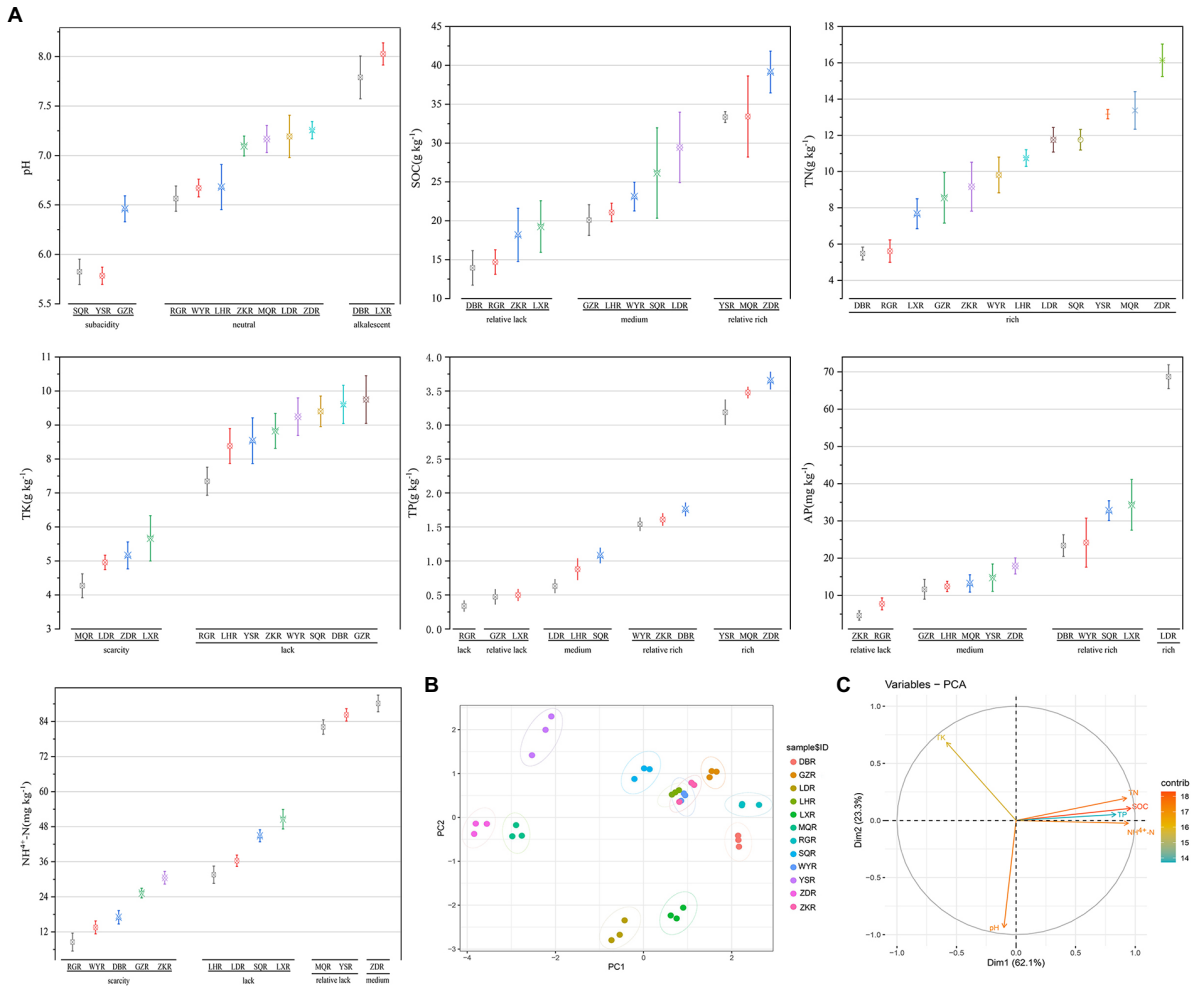
Comparative analysis of soil characteristics between different sites. R, Rhizosphere; following the same. **(A)** Grouping interval map of soil characteristics, including pH; SOC; TN; TP; TK; NH^4+^-N; AP. **(B)** Principal component analysis (PCA) of soil characteristics. **(C)** The contribution of the variables to the major components.

The soil characteristics of *F. przewalskii* in different habitat were summarized in Figure 2. Across sites, pH ranged from 5.78 to 8.03. Most soil samples were neutral (RG, WY, LH, ZK, MQ, LD, and ZD), a few were sub-acidic (SQ, YS, and GZ), and the remainder were alkaline (DB and LX sites) (Figure 2A). SOC, TN, TK, TP, AP, and NH^4+^-N were more variable among the 12 sites. TN was high at all sites, whereas TP and NH^4+^-N were low or scarce (Lin et al., 2017). When the rhizosphere soil environment was sub-acidic, C and N contents were most rich. PCA based on scaled soil characteristics indicated that soil properties varied greatly among habitats excluding ZK, WY, and LH (Figure 2B). The contributions of soil traits to major components of the PCA were differed, and the concentrations of N, C, and P were positively correlated with each other, and all three were significantly negatively correlated with K (Figure 2C).

### 3.3. Soil bacterial community diversity

**Figure 3:**
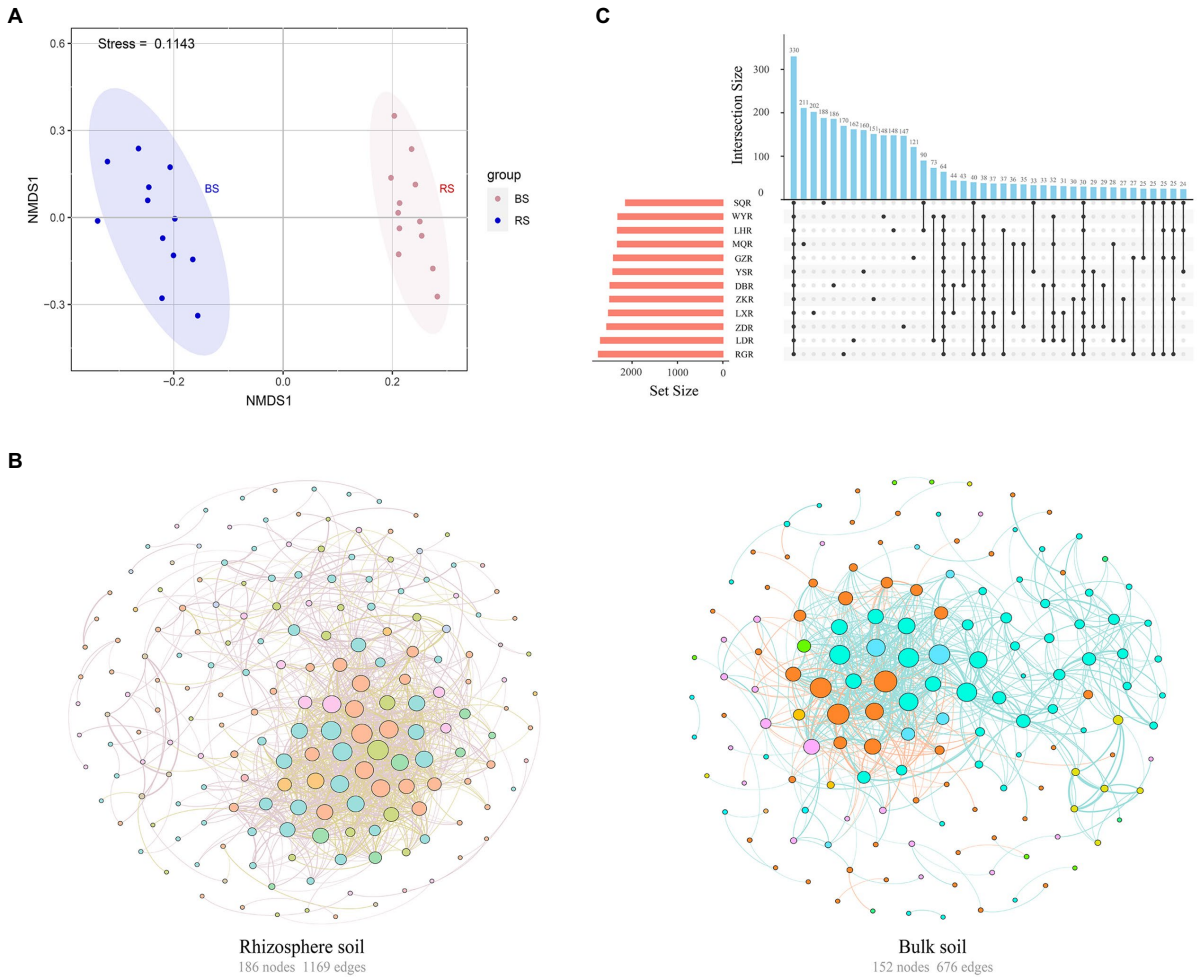
**(A)** Nonmetric multidimensional scaling (NMDS) of the bacterial communities in rhizosphere and bulk soils. RS, rhizosphere soil; BS, bulk soil. **(B)** Co-occurrence network of the bacterial communities in rhizosphere and bulk soils. The colors of edges represented positive correlation (SparCC *r* > 0.6, *p* < 0.01, pink or blue line) or negative correlation (SparCC *r* < −0.6, *p* < 0.01, yellow or orange line). The colors of nodes were based on the phylum to which OTU belong and the size of nodes was proportional to the number of connections (degrees). **(C)** Upset plot of the enriched tax in the rhizosphere soil among different sites.

Sequencing of 16S rRNA amplicons from 72 soil samples produced 2,549,758 paired-end Illumina MiSeq reads, with an average length of 418 bp. The sequences were clustered into 177,247 bacterial OTUs based on 97% sequence similarity. NMDS based on a Bray–Curtis dissimilarity matrix indicated that different soil samples were separated into two distinct groups of rhizosphere versus bulk, suggesting that the structure of the bacterial communities differed between rhizosphere and bulk soils (Figure 3A). Further analysis found that rhizosphere soil exhibited more complexity (number of nodes 186, edges 1,169, average degree 12.57) than bulk soil (number of nodes 152, edges 676, average degree 8.895) (Figure 3B). Similarly, the interaction between rhizosphere networks was more balanced, with 63.73% positive connections and 36.27% negative connections.

**Figure 4:**
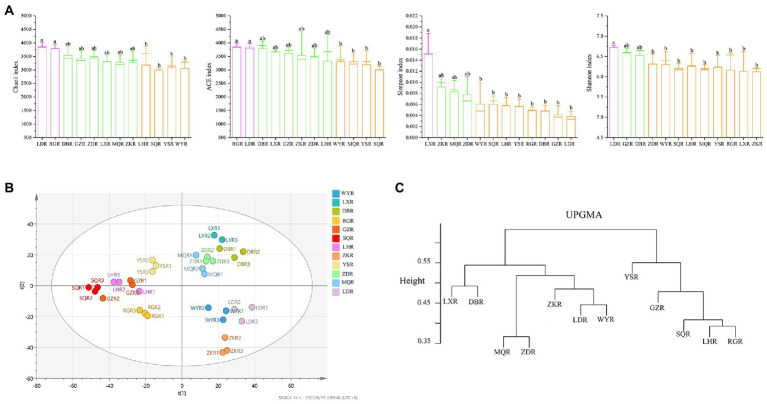
Diversity indices of rhizosphere soil bacterial communities. **(A)** Boxplots of the alpha-diversity indices, including Chao1 index; ACE index; Shannon index; Simpson index; different lowercase indicated significant differences between different regions (*p* < 0.05). **(B)** PLS-DA score map of the bacterial community. **(C)** UPGMA clusters of the different bacterial communities.

The similarity and specificity of OTUs in rhizosphere soils were displayed in Upset diagram (Figure 3C). The number of OTUs in RG site was the highest, while the SQ site had the lowest number. A total of 330 OTUs were found in all sites, and the numbers of specific OTUs for each site were 211 (MQR), 202 (LXR), 188 (SQR), 186 (DBR), 170 (RGR), 162 (LDR), 160 (YSR), 151 (ZKR), 148 (WYR), 148 (LHR), 147 (ZDR), and 121 (GZR). The alpha diversity of rhizosphere bacterial communities was compared at different sites *via* the Chao1 index, ACE index, Simpson index, and Shannon index (Figure 4A). The Chao1 and ACE indexes of abundance in LD and RG sites were significantly higher than WY, YS, and SQ (*p* < 0.05). Conversely, the Simpson index of diversity at LX was significantly higher than those at WY, SQ, LH, YS, RG, DB, GZ, and LD (*p* < 0.05), and the Shannon index at LD was significantly higher than those at ZD, WY, SQ, LH, MQ, YS, RG, LX, and ZK sites (*p* < 0.05).

PLS-DA analysis, a supervised method, showed that bacterial community composition varied between sites (Figure 4B). Samples with significant differences in community composition were clustered in different quadrants. A dendrogram constructed *via* UPGMA consistently showed that the sites were divided into two main clusters—bulk and rhizosphere soil (Figure 4C). In contrast, bacterial communities were more similar at the site level, particularly when comparing compositions at LX and DB sites, MQ and ZD sites, LD and WY sites, and LH and RG sites.

### 3.4. Soil bacterial community composition

**Figure 5:**
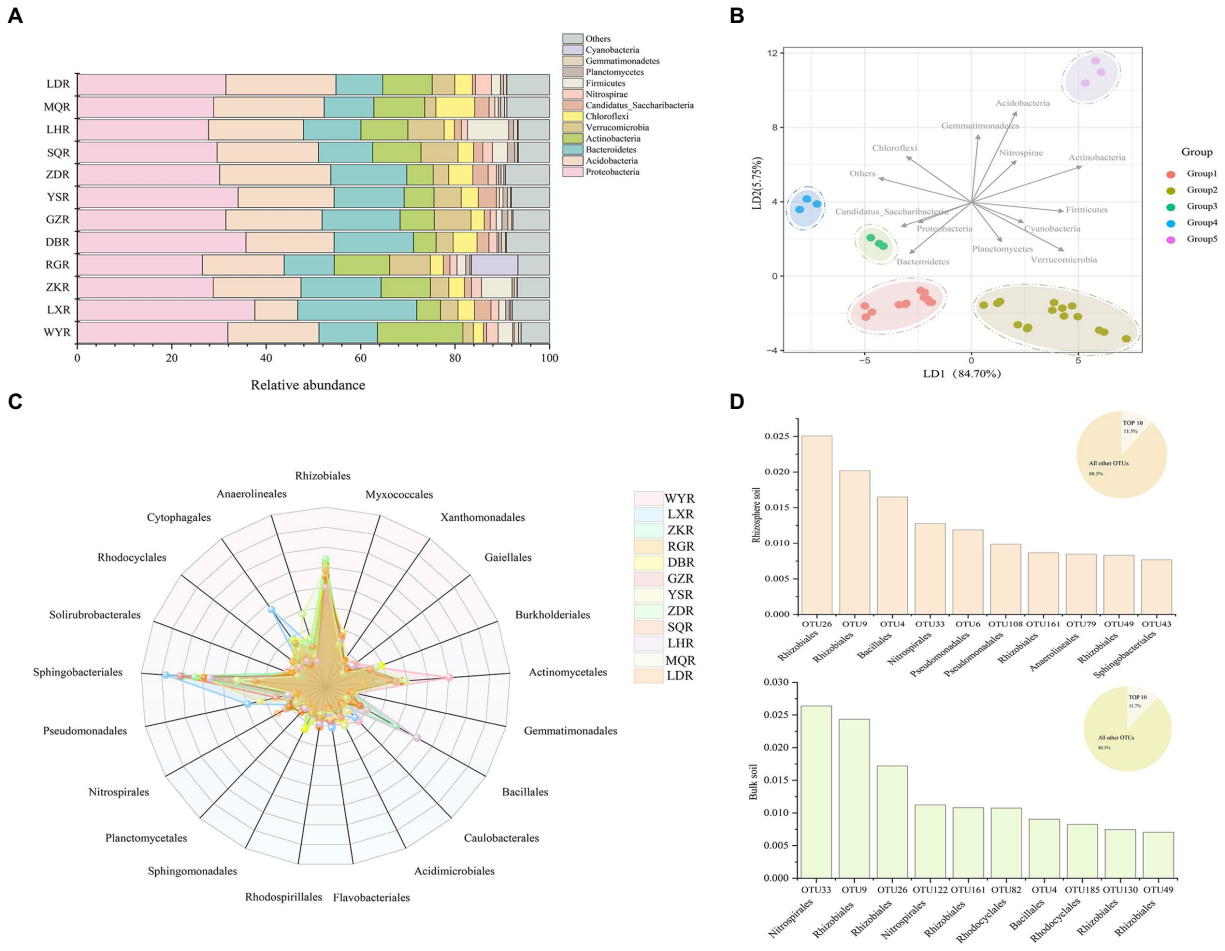
Bar chart of relative abundances of bacterial community **(A)** and Linear discriminant analysis (RDA) performed to distinguish the bacterial communities **(B)** at the phylum level; Radar plot displays the relative abundances of bacterial community **(C)** and Top 10 most abundant OTUs from rhizosphere soil and bulk soil at the order level **(D)**.

At the phylum level, Proteobacteria, Bacteroidetes, and Acidobacteria were the three most dominant phyla at all sites, with respective relative abundances of 26.47–37.61%, 10.53–25.22%, and 10.45–23.54%. We considered rare phyla to be those with relative abundances of <3%, which included Candidatus, Nitrospira, Planctomycetes, and Gemmatimonade (Figure 5A). Cyanobacteria and Firmicutes exhibited significantly higher relative abundance at RG and LH sites, respectively, and could be considered the dominant phyla at individual sites. LDA plot indicated that 12 sites were clustered into 5 groups (i.e., WY, DB, LD and LX sites were clustered into group 1, ZK, NR, GZ, LH and SQ sites were clustered into group 2, and ZD, YS, and MQ sites, respectively, for groups 3, 4, and 5) based on the relative abundance of phyla (Figure 5B). Specifically, Acidobacteria, Bacteroidetes, Actinobacteria, and Verrucomicrobia were critical for distinguishing rhizosphere bacterial communities between sites.

Differences in bacterial community composition were assessed at the order level. The most abundant orders were Rhizobiales, Sphingomonadales, and Actinomycetales which represented >60% of the relative abundance (Figure 5C). The relative abundances of Bacillales, Cytophagales, and Anaerolineales were significantly higher at LH, ZK, LX, and MQ. The top 10 most abundant OTUs in rhizosphere and bulk soil were compared, which together accounted for 11.5 and 11.7% of the respective total OTUs (Figure 5D). In rhizosphere soil, the top 10 OTUs were affiliated with six orders; Rhizobiales, Bacillales, Nitrospirales, Pseudomonadales, Anaerolineales, and Sphingobacteriales. In bulk soil they were affiliated with four orders, including Nitrospirales, Rhizobiales, Rhodocyclales, and Bacillales. This indicated that the bacterial communities in rhizosphere soil were more complex than those in bulk soil, consistent with results shown in Figure 3.

**Figure 6:**
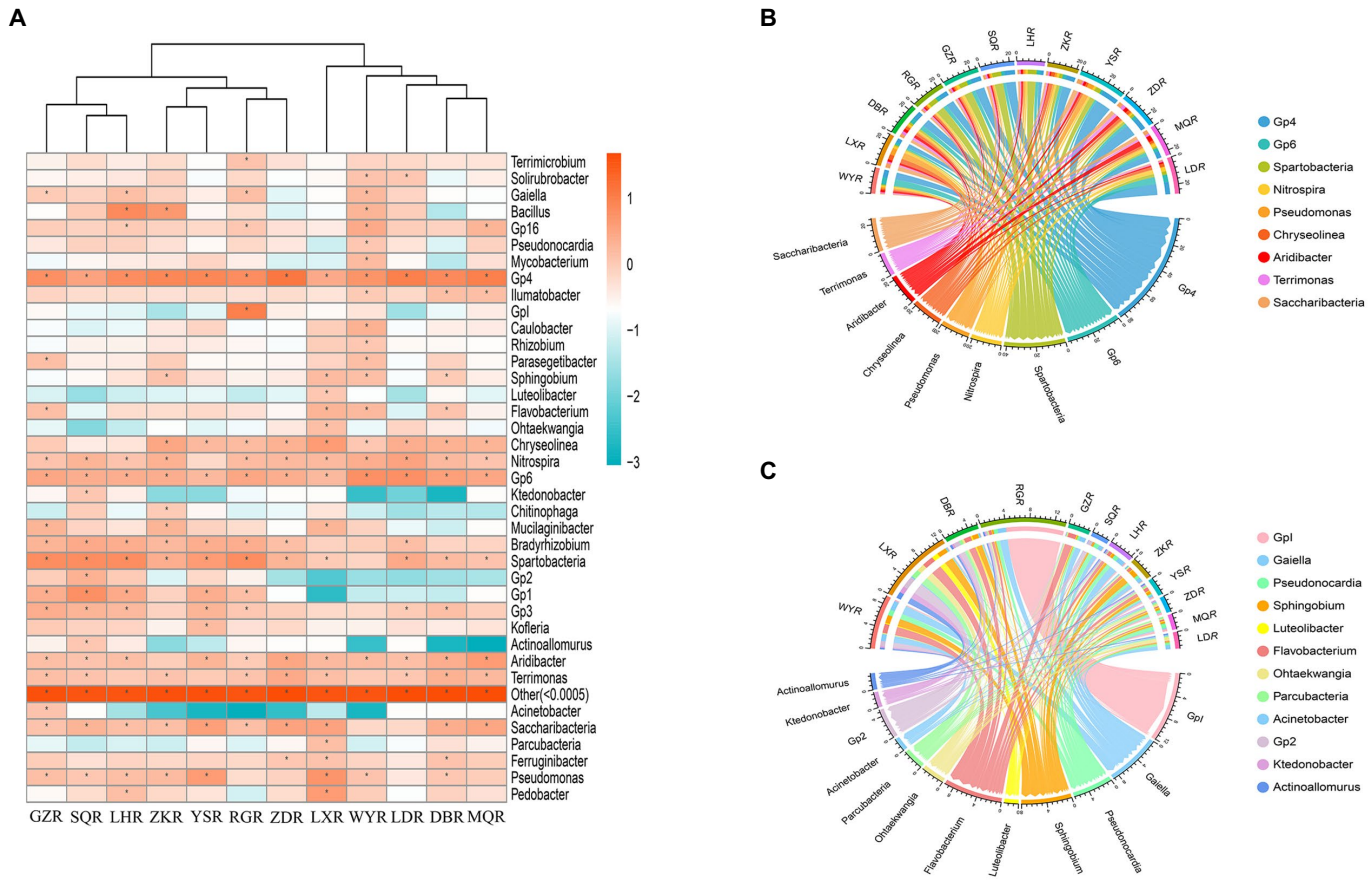
Community analysis at the genus level. **(A)** Heatmap of relative abundances, * represents relative abundance of greater than 1%. **(B)** Circos plot of relative abundances of common genera. **(C)** Circos plot of relative abundances of different genera.

We found that the specific distribution at the genus level was a major factor contributing to the differences of rhizosphere bacterial communities among sites (Figure 6A). When the relative abundance greater than 1% in each sample, we classified the common genera (Xie et al., 2021). In total, there were 9 common genera: *Gp4* (2.57–12.59%), *Spartobacteria* (1.02–6.46%), *Pseudomonas* (1.08–5.27%), *Gp6* (1.70–5.89%), *Aridibacter* (1.02–4.37%), *Chryseolinea* (1.12–3.98%), *Saccharibacteria* (1.23–3.08%), *Nitrospira* (1.23–3.39%), and *Terrimonas* (1.07–2.69%) (Figure 6B). There were also 12 different genera: *Acinetobacter* (GZ: 1.28%, others: 0.00–0.22%), *Pseudonocardia* (WY: 1.06%, others: 0.07–0.84%), *Gp2* (SQ: 2.10%, others: 0.005–0.66%), *Ktedonobacter* (SQ: 1.17%, others: 0.00–0.35%), *Actinoallomurus* (SQ: 1.17%, others: 0.00–0.38%), *GpI* (RG: 9.77%, others: 0.03–0.45%), *Parcubacteria* (LX: 1.43%, others: 0.03–0.53%), *Ohtaekwangia* (LX: 1.38%, others: 0.01–0.43%), *Luteolibacter* (LX: 1.13%, others: 0.03–0.25%), *Flavobacterium* (GZ, WY, and LX: 1.41–2.00%, others: 0.12–0.68%), *Sphingobium* (ZK, WY, and LX: 1.26–1.65%, others: 0.17–0.53%), and *Gaiella* (GZ, LH, NR, and WY: 1.03–1.73%, others: 0.11–0.77%) (Figure 6C).

### 3.5. Correlation analysis

**Figure 7:**
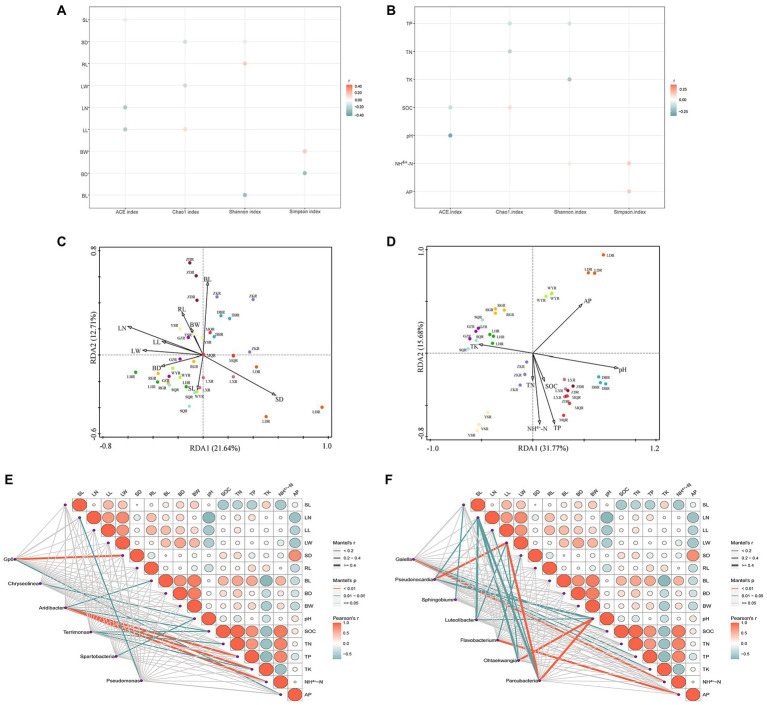
Correlation bubble plot between alpha diversity and plant phenotypic characteristics **(A)** or soil characteristics **(B)**. Redundancy analysis (RDA) performed to reveal the correlations between bacterial community structure and plant phenotypic characteristics **(C)** or soil characteristics **(D)**. Mantel analysis of plant phenotypic characteristics, soil characteristics with common genera **(E)** or different genera **(F)**. Color of the line represented the significance of the differences (*p*-value). Size of the line represented correlation coefficients (Mantel’s r).

In different habitats of *F. przewalskii*, plant phenotypic characteristics and soil characteristics were significantly correlated with the diversity, composition, and relative abundance of associated microorganisms, respectively (Figure 7).

Specifically, the ACE index was negatively correlated with stem length, leaf number, leaf length, pH, and SOC. The Chao1 index was positively correlated with leaf length and SOC, and negatively correlated with stem diameter, leaf width, TP, and TN. The Shannon index was positively correlated with stem diameter, root length, and NH^4+^-N, and negatively with bulb diameter and TK. The Simpson index was positively correlated with bulb length, NH^4+^-N, and AP, and negatively correlated with bulb horizontal and vertical dimensions.

Correlations between rhizosphere bacterial communities and plant phenotypic characteristics were shown in Figure 7C (axis1 = 21.64%, axis2 = 12.71%), and correlations between rhizosphere bacterial communities and soil characteristics were shown in Figure 7D (axis1 = 31.77%, axis2 = 15.68%). Using the RDA, we found that leaf number, stem diameter, bulb length, pH, TP, AP, NH^4+^-N, SOC, and TK contributed to the significant correlation with rhizosphere bacterial community structure (*p* < 0.05), and leaf number and pH were the most strongly contributing factors (Table 1). In contrast, soil characteristics were more related to rhizosphere microbial communities.

**Table 1:** The relative contribution of RDA analysis of plant phenotypic characteristics and soil characteristics to bacterial community structure.

Factor	Contribution %	*F*	*P*
Leaf number	14.1	1.5	0.004
Stem diameter	13.9	1.5	0.002
Bulb length	12.2	1.3	0.014
Bulb diameter	13.0	1.5	0.004
Stem length	10.9	1.2	0.066
Leaf width	9.6	1.1	0.292
Bulb width	9.2	1.0	0.426
Root length	8.7	1.0	0.558
Leaf length	8.3	0.9	0.656
pH	25.5	3.0	0.002
TP	14.9	1.8	0.002
AP	15.1	1.9	0.004
NH^4+^-N	14.4	1.8	0.002
SOC	11.8	1.5	0.002
TK	9.6	1.2	0.048
TN	8.5	1.1	0.244

Based on the analysis, 6 common microorganisms and 7 different microorganisms were selected at the genus level. Spearman analysis suggested that plant phenotypic characteristics were generally negatively correlated with soil characteristics (Figure 7E). Correlations with the special genera occurred in distinct patterns revealed by the Mental test (Figures 7E,F). All aboveground plant phenotypic characteristics were significantly correlated with different genera, particularly leaf width which was significantly correlated with *Pseudonocardia* and *Parcubacteria* (*p* < 0.01). Only leaf number, stem diameter, and bulb length were significantly correlated with common genera, with the stem diameter was most significantly correlated with *Aridibacter* (*p* < 0.01). All soil characteristics were significantly correlated with unique genera. Soil pH was more strongly correlated with unique genera than any other factor, whereas TN was only significantly correlated with common genera. Collectively, these results suggest a higher association between soil characteristics and bacterial community structure, consistent with the above analysis. Plant phenotypic characteristics on different genera were higher than common genera, while the reverse was observed with respect to soil characteristics.

## 4. Discussion

Due to its high medicinal value considerable interest is devoted to cultivating *F. przewalskii*. The natural *F. przewalskii* habitat is specialized, and the species is prone to soil-borne disease rendering cultivation more difficult (Cunningham et al., 2018). The associated microbiome can help host plants resist stress and adapt to the environment, promoting healthy growth (Koranda et al., 2011). Different habitats may exhibit diversity of microbiomes. In the current study, the diversity, composition, and structure of bacterial communities were compared at 12 in *F. przewalskii* growth sites, and plant phenotypic characteristics and soil characteristics at different sites based were compared with respect to microbial composition.

Previous studies indicate that plant development and secretory activities in root systems are intertwined, collectively forming a unique rhizosphere microbial community that differs from bulk microbial communities (Bulgarelli et al., 2013; Mendes et al., 2013). The results of the present study are consistent with this, and showed that the rhizosphere bacterial communities were distinctly separated from the bulk bacterial communities (Figure 3A). This was probably due to the strong recruiting effect of plant root on microbial communities (Chaparro et al., 2014). Co-occurrence network plots showed that the community structure of rhizosphere soil was more complex than that of bulk soil (Figure 3B), which is consistent with previous studies (Chen J. et al., 2020; de Albuquerque et al., 2022). This relates to the specific soil microhabitats in the rhizosphere.

Further, Chao1, ACE, Simpson, and Shannon alpha diversity indices varied weakly in rhizosphere soils between sites (Figure 4A), indicating that the rhizosphere bacterial community might be stable across sites (Martiny et al., 2013). Previous studies have hypothesized that environmental factors are critical for determining the structure of microbial communities (de Wit and Bouvier, 2006). Clustering results at the OTU level confirmed this, and sites with similar nutrient contents were more similar in bacterial community composition (Figure 4C). In the current study, the bacterial taxonomic compositions of *F. przewalskii* from different sites exhibited significant differences. Proteobacteria, Acidobacteria, Bacteroidetes, and Actinobacteria phyla formed the dominant microbiota despite habitat fluctuation (Figure 5A), which is consistent with previous studies (Shi et al., 2011; Jiao et al., 2022). Cyanobacteria was only the dominant phylum at RG site. Interestingly, previous studies have shown that Cyanobacteria can fix nitrogen (Elkoca et al., 2007), thus the distribution of Cyanobacteria may relate to the lower N content in RG site (Figure 2A). Most of the OTUs could be further classified at the order level in this study (Figure 5D). Among the top 10 most abundant OTUs, more than 0.06% of all the sequences were affiliated to Rhizobiales, which can provide specific secondary metabolites to plants, promoting nutrient cycling (Erlacher et al., 2015). Similarly, we found a proportion of both Pseudomonadales and Sphingobacteriales, which have highly antagonistic effects on specific bacterial and fungal pathogens, and may thus contribute to the stability of bacterial communities, ultimately promoting plant growth (Cernava et al., 2015; Larsbrink and McKee, 2020). To date, most research showed that plants can recruit beneficial microorganisms to their defense against environmental damage (Rudrappa et al., 2008; Raaijmakers et al., 2009), the active components in potential root secretory need further study.

Specific microorganisms were significantly enriched at each site, and we defined these enriched groups as core microorganisms (Zhang Y. et al., 2021). In the present study 21 core genera were detected, consisting of 9 common genera and 12 different genera (Figure 6). *Aridibacter*, *Pseudomonas*, *Spartobacteria*, *Gp6*, and *Gp4* were dominant at all sites, consistent with previous reports on rhizosphere soil (Liu W. et al., 2020; Jiao et al., 2022). Dominant microbial communities participate in soil nutrient cycling, and play important roles in ecosystem function regulation (Liu Y. et al., 2020). For example, *Aridibacter* and *Terrimonas* are significantly correlated with N and C due to their ability to degrade carbon and facilitate nitrogen removal (Sheu et al., 2010; Huber et al., 2017). Similarly, soil characteristics can influence the structure of microbial communities. RDA revealed that pH had the strongest effect on rhizosphere bacterial communities (Figure 7D). These results suggested that different habitats of *F. przewalskii* habitats can recruit different microorganisms to form a specific rhizosphere microbiome to adapt to soil microhabitats.

In addition, pH was significantly correlated with several dominant microorganisms (Figures 7E,F). These findings are consistent with previous studies suggesting that pH is the most influential environmental factor for soil microbial communities (Fierer and Jackson, 2006; Tripathi et al., 2018). *Spartobacteria* abundance was significantly correlated with pH. Interestingly, *Spartobacteria* is affiliated with Verrucomicrobia, which was shown to contain diverse acidophilic genera (Pol et al., 2007; Wang L. et al., 2021), thus presumably *Spartobacteria* might be acidophilic or acid tolerant. C and N content are also essential environmental factors that affect microbial communities (Yuan et al., 2014; Praeg et al., 2019), consistent with the results of the current study. In previous studies high SOC content led to significant enrichment of Proteobacteria (Fierer et al., 2008), thus the relative abundance of *Terrimonas* may be related to content of labile carbon substrates. Compared with other elements, P and K content were lower in the rhizosphere soil of *F. przewalskii* (Figure 2), although these could also affect bacterial communities (Figure 7). AP content was highest at the LD site, 10 times higher than at the RG site, which had the lowest AP content (Figure 2A). Further analysis revealed that the distribution of *GPI* was reversed (Figure 6A). This may be related to the role of *GPI* in degrading P, thought the mechanism needs involve requires further research. TK content was at an extremely low level in all soils, and was significantly negatively correlated with the Shannon index, and also with other nutrient contents (Figure 7B). The current study indicates that *F. przewalskii* is well adapted to a low potassium soil environment.

*Fritillaria przewalskii* was found at pH ranging from sub-acidity to alkalescency. The stem-leaf biomass of *F. przewalskii* was lowest in alkaline soil, especially at the DB site, which also had the lowest underground biomass (Supplementary Table S1). C and N content were also lowest in alkaline soil than in sub-acidic and neutral soil. These differences may be explained by the fact that C and N in soil are partly derived from stem and leaf litter which are influenced by root secretions (Postma et al., 2016; Liu Y. et al., 2020), while both above-and under-ground biomass was low at those sites. With the exception of K, overall nutrient availability was positively correlated with plant phenotypic characteristics (Figure 7E), indicating that higher C, N, and P content was beneficial to *F. przewalskii* growth. K was significantly negatively correlated with others (Figure 2C). Therefore, it is possible that excessive potassium content affects the growth of *F. przewalskii* and the uptake of other nutrients, though this premise warrants further study and discussion. As anticipated, there was strong relationship between plant phenotypic characteristics and microbial community structure (Figure 7). The factor that had the greatest effect was leaf number (Figure 7C), probably because leaves are a direct source of litter, and are important for shaping the soil environment (Ralte et al., 2005). As mentioned above, the structure and function of rhizosphere microorganisms directly affected soil nutrient availability, and therefore may also influence *F. przewalskii* growth. The current study was focused on the role played by beneficial microorganisms in the regulation of the soil environment and plant growth. However, harmful microorganisms are present in the rhizosphere environment (Dinesh et al., 2015). For example, continuous cropping of medicinal plants has limited the development of most Chinese medicine industries, and the accumulation of harmful microorganisms in the rhizosphere is regarded as a major cropping obstacle that we did not address (Xiao et al., 2019). Collectively, *F. przewalskii* plant, rhizosphere soil, and rhizosphere microbial communities form a complex system that is not yet understood, and considerable work is still required in the future.

## 5. Conclusion

The current study provides a comprehensive assessment of the bacterial communities of wild *F. przewalskii* in different habitats. Herein, we have reported that these bacterial communities varied between rhizosphere and bulk soil, and also between sites. Moreover, the diversity indexes varied little between habitats, and the microbial assembly was consistent with the trend of root-associated soil microhabitat distribution. The dominant phyla were Proteobacteria, Acidobacteria, Bacteroidetes, and Actinobacteria at 12 different habitats. Further, we highlighted the core microorganism species and functions, and concluded that microbiota prefer to enrich in a direction adapted to the environment of the host plant. Meanwhile, soil characteristics, particularly pH, had significant effects on bacterial communities, and it was surmised that these effect are derived from interactions between *F. przewalskii*, soil, and microorganisms. Leaf number was the most influential factor with respect to microbial communities, so we speculate that *F. przewalskii* may also change the microbial community *via* the accumulation of its own litters. These findings enhance our understanding of *F. przewalskii* root-associated soil microhabitats. In addition, the study also suggests that sub-acidic soil more suitable for the growth of *F. przewalskii*. Whether excessive potassium affects the rhizosphere microbial communities of *F. przewalskii*, and whether this has negative effects on *F. przewalskii* growth warrant future investigation.

## Data availability statement

The datasets presented in this study can be found in online repositories. The names of the repository/repositories and accession number(s) can be found at: NCBI – PRJNA901426.

## Author contributions

ZC and LJ conceived and designed the experiments. ZC, RL, FL, HW, and CC performed the experiments. ZC and RL analyzed the data and wrote the manuscript. ZC, LJ, YM, ZW, and YW revised the manuscript. All authors contributed to the article and approved the submitted version.

## Funding

This work was funded by the “Double First-Class” research key project of Gansu (GSSYLXM-05), ecological planting and quality assurance project of genuine medicinal materials (2020-No. 153), and Gansu province higher education innovation fund project (2021A-083).

## Conflict of interest

The authors declare that the research was conducted in the absence of any commercial or financial relationships that could be construed as a potential conflict of interest.

## Publisher’s note

All claims expressed in this article are solely those of the authors and do not necessarily represent those of their affiliated organizations, or those of the publisher, the editors and the reviewers. Any product that may be evaluated in this article, or claim that may be made by its manufacturer, is not guaranteed or endorsed by the publisher.
