# Supplementary material for: Structural characteristics and diversity of the rhizosphere bacterial communities of wild Fritillaria przewalskii Maxim. in the northeastern Tibetan Plateau
Source: Front Microbiol. 2023 Feb 17;14:1070815. doi: 10.3389/fmicb.2023.1070815 (PMC9981654; doi:10.3389/fmicb.2023.1070815)
Supplement: Supplementary file 9 [file Table_1.DOC]

**Supplementary Table 1** The plant phenotypic characteristics in the different sites

| Sampling site | Aboveground part | | | | | Underground part | | | |
| --- | --- | --- | --- | --- | --- | --- | --- | --- | --- |
|  | Stem length (cm) | Leaf number (sc) | Leaf length (mm) | Leaf width (mm) | Stem diameter (mm) | Root length (cm) | Bulb length (mm) | Bulb diameter (mm) | Bulb weight (g) |
| WY | 42.30±8.08ab | 6.33±0.67ab | 72.03±16.87a | 4.67±1.03ab | 2.03±0.09a | 4.47±0.39a | 11.04±0.85a | 12.31±1.54ab | 0.93±0.28bc |
| LX | 37.43±0.72ab | 4.00±0.58b | 39.85±1.71b | 2.80±0.39b | 1.56±0.10a | 2.79±0.34a | 9.17±0.38ab | 10.42±1.40ab | 0.87±0.04bc |
| DB | 25.63±2.82b | 5.00±0.00b | 31.56±3.87b | 2.82±0.38b | 1.66±0.15a | 3.60±1.37a | 6.83±0.60b | 5.77±0.05c | 0.15±0.03c |
| RG | 34.95±2.34ab | 6.50±0.29a | 54.33±2.03ab | 4.63±0.48ab | 1.66±0.05a | 3.14±0.74a | 7.89±0.72ab | 8.60±0.19b | 0.31±0.05c |
| GZ | 31.50±4.40ab | 5.60±0.40ab | 66.50±10.06ab | 4.05±0.38ab | 1.65±0.10a | 4.67±1.37a | 9.16±0.94ab | 9.71±0.99ab | 0.55±0.14bc |
| SQ | 29.10±5.16b | 6.20±0.37ab | 59.28±10.47ab | 4.82±0.63a | 1.82±0.12a | 2.91±0.22a | 8.38±0.56ab | 10.22±0.75ab | 0.52±0.09bc |
| LH | 32.10±3.82ab | 6.20±0.37ab | 57.88±14.27ab | 4.74±0.40ab | 1.86±0.12a | 3.27±0.60a | 10.72±0.53a | 13.52±0.78a | 1.21±0.19ab |
| ZK | 45.40±9.55a | 5.67±0.67ab | 77.00±22.13a | 4.98±0.83a | 1.70±0.34a | 3.13±0.10a | 12.71±3.21a | 10.71±2.12ab | 1.46±0.63ab |
| YS | 28.06±2.26b | 6.20±0.20ab | 62.76±5.04ab | 3.37±0.28ab | 1.53±0.11a | 4.37±0.79a | 9.84±0.38ab | 8.25±0.50bc | 0.40±0.04c |
| ZD | 21.88±4.27b | 6.00±0.32ab | 62.92±8.88ab | 3.94±0.81ab | 1.82±0.09a | 3.91±0.84a | 12.24±1.08a | 10.36±0.97ab | 0.71±0.14bc |
| MQ | 26.12±4.24b | 5.40±0.40ab | 40.02±5.44b | 4.16±0.94ab | 1.75±0.29a | 3.05±0.43a | 12.93±4.41a | 13.08±4.23ab | 1.63±1.30a |
| LD | 29.77±2.45b | 4.33±0.67b | 39.20±5.31b | 2.83±0.27b | 2.49±0.31a | 2.71±0.41a | 8.48±0.29ab | 7.90±0.83bc | 0.26±0.03c |

All data are presented as mean ± SD. Different letters indicate significant differences (*p*<0.05) among different sampling sites
